# Transcranial Electric Current Stimulation During Associative Memory Encoding: Comparing tACS and tDCS Effects in Healthy Aging

**DOI:** 10.3389/fnagi.2020.00066

**Published:** 2020-03-17

**Authors:** Katharina Klink, Jessica Peter, Patric Wyss, Stefan Klöppel

**Affiliations:** University Hospital of Old Age Psychiatry and Psychotherapy, University of Bern, Bern, Switzerland

**Keywords:** episodic memory, associative memory, healthy aging, transcranial direct current stimulation, transcranial alternating current stimulation, frontal cortex, ventrolateral prefrontal cortex

## Abstract

Associative memory is one of the first cognitive functions negatively affected by healthy and pathological aging processes. Non-invasive brain stimulation (NIBS) techniques are easily administrable tools to support memory. However, the optimal stimulation parameters inducing a reliable positive effect on older adult’s memory performance remain mostly unclear. In our randomized, double-blind, cross-over study, 28 healthy older adults (16 females; 71.18 + 6.42 years of age) received anodal transcranial direct (tDCS), alternating current in the theta range (tACS), and sham stimulation over the left ventrolateral prefrontal cortex (VLPFC) each once during encoding. We tested associative memory performance with cued recall and recognition tasks after a retention period and again on the following day. Overall, neither tDCS nor tACS showed effects on associative memory performance. Further analysis revealed a significant difference for performance on the cued recall task under tACS compared to sham when accounting for age. Our results suggest that tACS might be more effective to improve associative memory performance than tDCS in higher aged samples.

## Introduction

The decline of episodic memory performance is one of the most prominent changes in cognitive function in aging (Grady, 2012). According to the associative memory deficit hypothesis, this decline is specific to difficulties in connecting unrelated units into one cohesive episode (Naveh-Benjamin et al., 2003). Associative memory is the memory of both item-context and item-item associations (Wang and Cabeza, 2016). Further, aging negatively affects this ability to form and retrieve links between different chunks of information given by items and their contexts, e.g., spatial location and color of an item. The same applies to connecting two different items into one unit, e.g., a name and a face (Old and Naveh-Benjamin, 2008). The association of names or occupations with faces is a common test of associative memory (Vannini et al., 2011). Using the association of occupations and faces creates a broader semantic context, that can be used as a cue for later retrieval (Duss et al., 2011).

Successful associative memory encoding and retrieval, the formation of new memory contents and their recollection, depend on the interaction of the medial temporal lobe with widespread neocortical regions, including the prefrontal cortex (PFC; Ranganath, 2010). For the rapid encoding of flexible associations (Henke, 2010), the hippocampus (HC) is a critical brain structure (Eichenbaum et al., 2007). HC functions become progressively impaired with age (Raz et al., 2005), which is also reflected in age-related reductions of HC activity specific for associative compared to item memory (Dennis et al., 2008). On the other hand, older adults’ associative memory performance benefits from increased activity in the PFC (Bangen et al., 2012). A causal link of associative memory formation to cortico-hippocampal network activity has been demonstrated by a study conducting multiple sessions of individually targeted repeated transcranial magnetic stimulation over the left lateral parietal cortex: it improved associative memory performance by increasing functional connectivity between cortical regions and the HC (Wang et al., 2014).

Non-invasive brain stimulation (NIBS) techniques have the potential to alter large-scale brain network activity in order to support and maintain cognitive functioning affected by aging processes (Gutchess, 2014). An extensive review and meta-analysis of NIBS effects on cognitive function in normal and pathological aging showed small to medium positive effects of the technique (Hsu et al., 2015). NIBS methods like transcranial direct and alternating current stimulation are inexpensive and safe to use if safety guidelines are followed (Woods et al., 2016). The underlying physiological mechanisms of transcranial direct current stimulation (tDCS) differ from those underlying transcranial alternating current stimulation (tACS). tDCS modulates the excitability thresholds of the neuronal membrane in a polarity-specific manner by inducing either depolarization or hyperpolarization (Antonenko et al., 2016). Thus, it directly affects the firing rate of neurons (Reato et al., 2010). tACS, on the other hand, up- and down-regulates the firing rate of neurons in an oscillatory manner without changing the average firing rate over a longer time interval. Therefore, stimulation at the frequency of endogenous oscillations mainly affects the spike timing of the neurons (Reato et al., 2010). Although NIBS methods seem to be a promising tool to maintain or enhance older adults’ episodic memory performance, most of the previous tDCS studies on episodic memory have been conducted in healthy young adults. Results of studies in young populations have been mixed (Galli et al., 2019) and cannot easily be transferred to older adults, since the stimulation sites and intensities that modulate memory performance in young adults might not improve or even disturb older adults’ memory performance (Perceval et al., 2016). Only a handful of studies investigated the effects of tDCS on episodic memory encoding in healthy elderly subjects: anodal tDCS applied over the right temporoparietal cortex during memory encoding significantly improved delayed recall performance on an associative object-location learning task (Flöel et al., 2012). Another study applying anodal tDCS over the left VLPFC during word learning resulted in increased discrimination accuracy in a recognition task (Medvedeva et al., 2019). Anodal tDCS applied over the left dorsolateral PFC (DLPFC) during the encoding of a list of words resulted in enhanced performance on a free recall task (Sandrini et al., 2016). Yet another study using the same stimulation site during face-name associative memory encoding found no stimulation effect on memory performance, neither in a cued recall nor a recognition task (Leach et al., 2019; Peter et al., 2019). To our knowledge, no studies investigating the effect of tACS on episodic memory encoding in healthy older adults have been published so far. However, one pilot study applying 20 min of tACS in the theta frequency range over the temporoparietal cortex of healthy elderly participants simultaneous to an implicit language learning task found memory enhancing effects (Antonenko et al., 2016). Theta tACS is regularly investigated in connection with memory related tasks (Antal and Paulus, 2013).

Since inter-individual differences of the susceptibility to NIBS have been shown (Jamil et al., 2017), we aimed to investigate the intra-individual differences in effects of tDCS and tACS on associative memory formation using a crossover within-subject and sham-controlled design. We administered the stimulation to the left VLPFC because functional magnetic resonance imaging (fMRI) studies have repeatedly shown that tasks involving semantic processing specifically activate the VLPFC (Thompson-Schill et al., 2005). Additionally, a study applying anodal tDCS over the VLPFC while simultaneously measuring fMRI improved older adults performance up to the level of younger adults on a semantic word generation task, while reducing task-related hyperactivity in bilateral prefrontal cortices and inducing a more youth-like functional connectivity pattern in the elderly participants (Meinzer et al., 2013). To increase stimulation focality, we used different electrode sizes for anode and cathode, following the stimulation protocol used by Meinzer et al. (2012). We applied 2 mA of tDCS because stimulation at this intensity seems to yield more reliable effects than tDCS at 1 mA—at least in cortical excitability over the motor cortex (Ammann et al., 2017). For the tACS stimulation protocol, we decided to apply stimulation in the theta frequency range, since slow oscillations underlie cognitive functions (Antal and Paulus, 2013). In particular, the theta frequency spectrum in human electroencephalography (EEG) has been associated with episodic memory processes, event-related theta synchronization during the encoding of new information is proposed to reflect theta activity that is induced into the cortex *via* cortico-hippocampal feedback loops (Klimesch, 1999). We applied tACS at a lower intensity of 1 mA to avoid retinal phosphenes, since the bigger electrode was placed in close proximity to the right eye over the supraorbital cortex (Schutter and Hortensius, 2010).

In the present study, we aimed to answer the question whether tDCS and tACS are able to improve associative episodic memory encoding in healthy elderly adults. Additionally, we wanted to investigate whether one of the methods is superior to the other. We hypothesized that both tDCS and tACS applied during the encoding of face-occupation pairs would improve cued recall and recognition task performance, compared to sham stimulation.

## Materials and Methods

### Study Procedure

**Figure 1:**
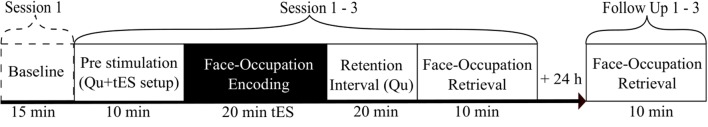
An overview of the study procedure. Each of the three sessions consisted of encoding, consolidation, and retrieval. The retrieval was repeated after 24 h *via* an online survey. The baseline measurement (cognitive screening, paired associates test) was completed in the first session. Transcranial electric stimulation (tES) was applied during encoding. Different questionnaires (Qu) were given before the encoding and during the retention phase.

In this double-blinded, sham-controlled, cross-over study each participant attended three sessions at the University Hospital of Old Age Psychiatry and Psychotherapy in Bern. Every session consisted of an encoding, retention, and retrieval phase, while only the first session included a cognitive screening (MoCA, Nasreddine et al., 2005; Benton Test, Benton Sivan and Spreen, 2009) and an additional paired associates test as a baseline measurement for associative memory performance (Figure 1). Additionally, the retrieval was repeated the following day at home using either an online survey or a paper-pencil questionnaire to assess any consolidation effects overnight. The sessions were always scheduled in the mornings at least 72 h apart to avoid stimulation carry-over effects (Hoy et al., 2015) as well as day time effects (Li et al., 2015). The order of stimulation methods was randomized for the three conditions (tACS/tDCS/sham). Data management as well as randomization was carried out using REDCap electronic data capture tools hosted by the Clinical Trials Unit of the University of Bern (Harris et al., 2009).

### Participants

**Figure 2:**
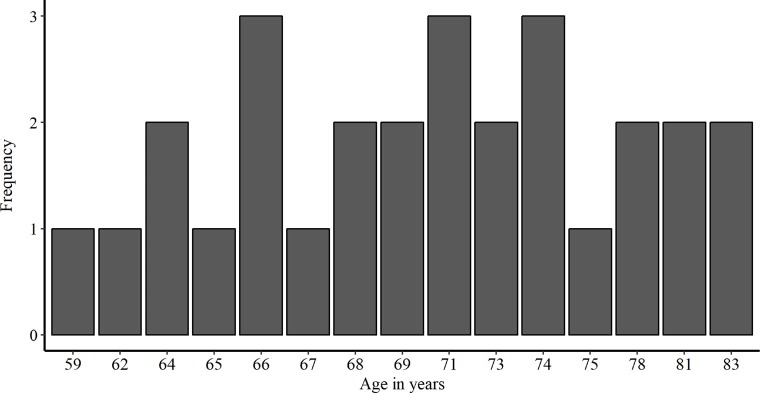
Histogram showing the frequency distribution of age in years in the study population.

We recruited 28 healthy older adults from Bern, Switzerland and surrounding communities (16 females and 12 males; age mean ± SD, 71.18 + 6.42 years; range, 59–83 years). A preliminary telephone screening with each subject ensured that all inclusion criteria for transcranial electric stimulation (tES) applications were met (i.e., no pacemakers, no metal implants, no abrasions on the scalp, no prior brain injuries/surgeries or past epileptic seizures). Additionally, we excluded smokers and non-German speakers. To assure cognitive functioning within age-related norms, all participants underwent a cognitive screening (MoCA, Nasreddine et al., 2005). The study was approved by the local Ethics Committee. Prior to testing, we obtained written informed consent from all participants. The demographic information of the participants enrolled in this study is presented in Table 1. Additionally, a histogram of the age distribution is shown in Figure 2.

**Table 1:** Demographic data of the sample and baseline performance prior to stimulation.

	Mean (*SD*)	Range
Age (years)	71.18 (6.42)	59–83
Years of education	14.79 (3.4)	7–22
MoCA (0–30)	27.25 (2.17)	23–30
GDS (0–15)	0.79 (1.13)	0–4
PA delayed recall (0–18)	13.11 (2.74)	8–18
BT-A (0–10)	5.96 (1.35)	3–8

*Note: BT-A, Benton Test A (Benton Sivan and Spreen, 2009); GDS, Geriatric Depression Scale (Yesavage et al., 1983); MoCA, Montreal Cognitive Assessment (Nasreddine et al., 2005); PA, paired associates test (Petermann and Lepach, 2012; Spaan, 2016)*.

### Experimental Schedule

#### Baseline

Only during the first session participants performed the following baseline measurement: first, they completed a cognitive screening (MoCA, Nasreddine et al., 2005), followed by a paired associates test (PA; Petermann and Lepach, 2012; Spaan, 2016). Between encoding/immediate recall and the delayed recall of the PA test, the Benton Test (Benton Sivan and Spreen, 2009) was performed.

#### Pre-stimulation

**Figure 3:**
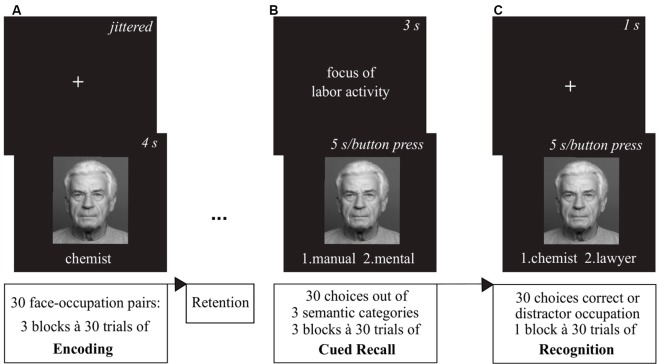
Overview of the associative memory task. **(A)** During the encoding phase, participants were presented 30 face-occupation pairs in a randomized order with each pair shown three times.** (B)** After the retention interval, the cued recall task showed the faces as cue and participants were asked to decide which of two choices out of three categories (1. *Education*/1st choice—university degree, 2nd choice—apprenticeship; 2. *Maximum Income*/1st choice—above average, 2nd choice—average; 3. *Focus of Labor Activity*/1st choice—mental, 2nd choice—manual) the previously encoded occupation of the depicted person matched. **(C)** The subsequent recognition task again presented the faces as cues and participants had to choose between the correct and a distractor occupation. Images of faces were derived from the FACES database (Ebner et al., 2010).

For all three sessions participants completed the pre-stimulation positive and negative affect scale (PANAS; Watson et al., 1988). Afterwards, they were given a short training version of the face-occupation task, where they encoded three face-occupation pairs (Figure 3A). Following encoding they practiced the cued recall (Figure 3B) and the recognition task (Figure 3C) with feedback, to make sure each task was understood properly. Following the mounting of stimulation electrodes, participants were given approximately 1 min to get accustomed to the tingling sensation of the tES ramp-up phase.

#### Encoding

During encoding, we presented the participants with a total of 30 face-occupation pairs repeated in three blocks. The order of the stimuli was randomized within each block. Faces were presented in color with the occupation written underneath in white against a black background and shown for 4 s. Preceding each face-occupation pair was a white fixation cross centered on a black background. The presentation duration was jittered between 2.8–9.8 s with an average length of 7 s. After completion of 20 min of encoding with concurrent stimulation, the electrodes were taken off.

#### Retention

In the following retention phase of approximately 20 min participants were administered questionnaires assessing adequate blinding, immediate tES side effects and the post-stimulation PANAS (Watson et al., 1988).

#### Retrieval Sessions

During the retrieval phase participants were administered a cued recall task first. The previously encoded faces served as a cue for the semantic categorization task. Participants were asked to decide which of two choices out of three categories (1. *Education*/1st choice—university degree, 2nd choice—apprenticeship; 2. *Maximum Income*/1st choice—above average, 2nd choice—average; 3. *Focus of Labor Activity*/1st choice—mental, 2nd choice—manual) the previously encoded occupation of the person matched *via* button press. Following a fixation cross shown for 1 s, the category was presented for 3 s. Then, the face with the two choices written underneath was shown for a maximum of 5 s or until an answer was given. After completion of the 30 cued recall runs, a recognition task was presented to the participants. Again, a fixation cross shown for 1 s was followed by a face cue with two choices written underneath shown for a maximum of 5 s or ended *via* button press. In the recognition task participants had to decide between the correct and a distractor occupation. All distractor occupations were selected from the same set of 30 occupations encoded before.

#### Retrieval Follow-Ups

On the day following each session, participants were again asked to answer the tES side effect questionnaire and presented the cued recall as well as the recognition tasks using either an online survey provided by REDCap or a paper-pencil version of the survey.

### Transcranial Electric Current Stimulation

Transcranial electric current stimulation was delivered using electrodes inserted in saline-soaked sponges (neuroConn DC-Stimulator Plus; neuroCare Group GmbH, Munich, Germany). Electrode positions were individually defined according to the 10–20 EEG system using the same positioning method and electrode sizes as in the study by Meinzer et al. (2012): following their protocol we determined the intersection of T3-F3 and F7-C3, as well as the midpoint between F7-F3 and positioned the anodal/target electrode (5 × 7 cm^2^) at the center of a line connecting those two points aiming for the left VLPFC as stimulated area. We positioned the cathodal/return electrode over the right supraorbital area (10 × 10 cm^2^). Twenty minutes of tES was applied with the different conditions being randomized in a cross-over fashion. tDCS was delivered with a current of 2 mA (current density = 0.06 mA/cm^2^), while tACS was delivered with 1 mA (current density = 0.03 mA/cm^2^). For the sham condition half of the participants received sham-tACS with the current ramped up to 1 mA, the other half of participants received sham-tDCS with the current ramped up to 2 mA. For both sham-variations, the current was turned off after 30 s. All conditions had a 15 s fade-in/out period.

### Stimuli for Associative Memory and Baseline Tasks

For the baseline paired associates test we retrieved 18-word pairs from the 4th German edition of the Wechsler Memory Scale (Petermann and Lepach, 2012) and a publication evaluating the diagnostic accuracy of paired-associate learning formats (Spaan, 2016). For the associative memory task, we created a face-occupation task (Duss et al., 2011). In an online-survey 30 German-speaking participants [21 females and 9 males, age mean ± SD, 29.79 + 5.13 years; range, 21–42 years, years of education median (range), 18 (13–21) years] rated occupations according to three different categories (1. *Education*/1st choice—university degree, 2nd choice—apprenticeship; 2. *Maximum Income*/1st choice—above average, 2nd choice—average; 3. *Focus of Labor Activity*/1st choice—mental, 2nd choice—manual). We used the 90 out of 128 occupations from a Swiss internet service informing about possible career paths^1^ that attained the most congruent choices on the online survey. Ninty color photographs of faces were derived from the FACES database (Ebner et al., 2010). All faces showed a neutral expression and were equally balanced over gender and age groups (young, middle-aged, elderly). The same three sets of 30 stimuli were used for all participants in the same order over the consecutive sessions. Each set consisted of 15 academic and 15 non-academic occupations (for a complete list of occupations used in the task, see Supplementary Table S1).

### Statistical Analysis

We defined the outcome for associative memory retrieval as the total number of correct answers given in the cued recall and the recognition tasks, respectively. We analyzed the performance of sessions and follow-ups independently because the response time was limited only during the sessions. We analyzed the data using linear mixed effect models (LMEM) fit by maximum likelihood (Baayen et al., 2008; Barr et al., 2013), enabling us to include age as a continuous covariate. We wanted to include age as a covariate since our study sample showed a large age range between 59 and 83 years. In longitudinal studies assessing episodic memory performance over the life span, a clear performance drop can be found between the ages of 60–65 years, continuing to decline with increasing age (Nyberg et al., 2012). We analyzed the data using the statistical program R (R Core Team, 2018), fitting all models with the package lme4 (Bates et al., 2015). As a first step we defined a full model including the following variables of interest and covariates: for session and follow-up data, we included the *stimulation method* as the main fixed effect of interest. Additionally, we entered an interaction term for *stimulation method* and *age*. As covariates, we included baseline associative memory performance in the delayed recall of the paired associates test, MoCA values, gender, years of education, sessions and age in the fixed effects structure of the model. Only for the models of follow-up data, we added a covariate for performance at the preceding session. All continuous covariates were centered to keep the random variance component and fixed-parameter estimates informative (Judd et al., 2017). All the effects were tested using likelihood ratio tests. We compared the full model with a reduced model for the random and fixed effects structure of the models.

For the random effects structure of models for the *session*, we included a by-subject random intercept. We included a by-subject random slope in addition to the by-subject random intercept in the models of follow-up performance to control for repeated administration of the same items.

Last, we report test values and significance levels of analysis of variance based on Satterthwaite’s degrees of freedom approximation applied by the lmerTest R package (Kuznetsova et al., 2017) for the final models. We used visual inspections (q-q-plots of residuals and estimates) to check for the assumed normal distribution.

## Results

Our key goal was to evaluate the efficacy of the NIBS techniques anodal tDCS and theta tACS for associative memory enhancement in healthy elderly adults. We used a cued recall and a recognition task to assess memory performance at different time points. We report results separately for cued recall and recognition tasks as well as for session and follow-up time points. For a comprehensive overview of the reduced model estimates and test values see Supplementary Tables S2, S3.

### Cued Recall Task

#### Sessions

**Figure 4:**
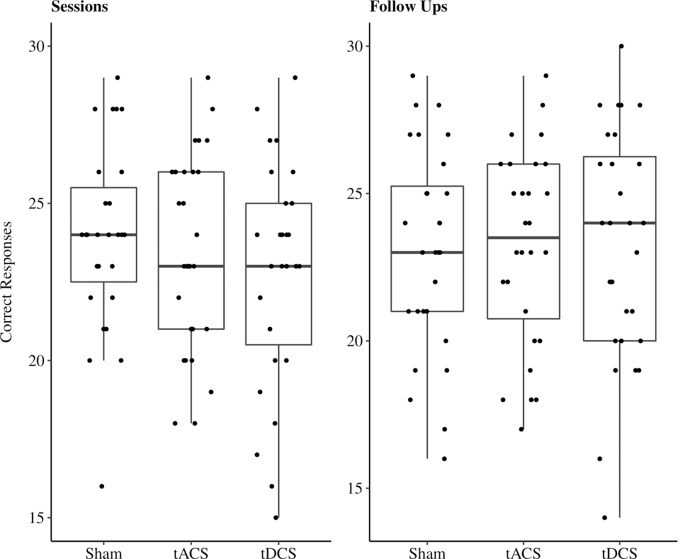
Boxplots with individual data points of sum of correct responses for the cued recall task jittered and divided by stimulation method. Abbreviations: tACS, alternating current stimulation; tDCS, direct current stimulation; Sham, control condition.

**Figure 5:**
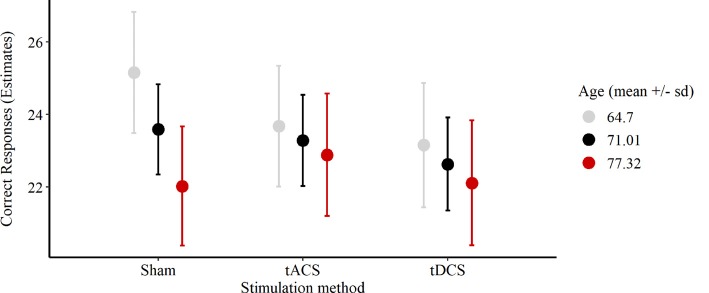
Plot of the interaction effect of age and stimulation condition for the cued recall task outcome during the sessions. Estimates of the number of correct responses expected for the mean age (respective one *SD* below and above) under each stimulation method are depicted. Error bars are 95%-confidence intervals around the estimates. Abbreviations: tACS, alternating current stimulation; tDCS, direct current stimulation; Sham, control condition.

In the cued recall task, on average 23.11 out of 30 possible correct responses were given over all three sessions. We excluded two participants’ responses due to performance at chance level (33%, 40%). In 11% of all sessions performances reached a high accuracy level between 28 and 30 correct responses (Figure 4). The reduced LMEM for the cued recall data included a by-subject random intercept ($X_{\left(1\right)}^{2}$ = 17.78, *p* < 0.001) as random effects structure. For the fixed effects structure interaction effect for age and stimulation method ($X_{\left(4\right)}^{2}$ = 18.82, *p* < 0.001) significantly increased the model fit. Additionally, we included the covariates years of education ($X_{\left(4\right)}^{2}$ = 5.7, *p* < 0.05) and session ($X_{\left(2\right)}^{2}$ = 12.95, *p* < 0.01) in the final model. The fixed effect structure of the final model explained 32.6% of the variance (marginal *R*^2^) and total variance explained by fixed and random effects was 69% (conditional *R*^2^; Nakagawa and Schielzeth, 2013). The LMEM revealed no significant main effect for stimulation (*F*_(2,48)_ = 1.52, *p* = 0.23). The main effect for age (*F*_(1,25)_ = 3.15, *p* = 0.09) and the interaction effect between age and stimulation (*F*_(2,48)_ = 2.84, *p* = 0.07) showed a trend towards significance. Additionally, a significant effect of session sequences was revealed (*F*_(2,48)_ = 6.55, *p* < 0.05). We tested fixed age effects for every stimulation separately using conditional *t*-tests. Under sham stimulation performance significantly decreased with increasing age [*β* = −1.58, 95%-CI: (−2.64, −0.52), *t*_(47)_ = −2.8, *p* < 0.01], while there was no significant age effect for tACS [*β* = −0.4, 95%-CI: (−1.47, 0.67), *t*_(48)_ = −0.7, *p* = 0.49] and tDCS [*β* = −0.52, 95%-CI: (−1.62, 0.58), *t*_(51)_ = −0.9, *p* = 0.37]. Moreover, we compared the fixed age effects between the stimulation conditions using conditional *t*-tests, revealing a significant difference between the age effect under tACS and the age effect under sham stimulation [*β* = 1.18, 95%-CI: (0.16, 2.2), *t*_(48)_ = 2.17, *p* < 0.05, Figure 5], whereas there was no significant difference between tDCS and sham [*β* = 1.05, 95%-CI: (−0.02, 2.12), *t*_(48)_ = 1.9, *p* = 0.07, Figure 5]. In session three participants performed significantly better compared to session one [β = 1.31, 95%-CI: (0.27, 2.35), *t*_(48)_ = 2.36, *p* < 0.05), while performance during session two was not significantly different from session one [*β* = −0.72, 95%-CI: (−1.68, 0.68), *t*_(48)_ = −1.3, *p* = 0.2]. For additional plots showing cued recall performance distributions over age and baseline performance groups see Supplementary Figures S1, S2.

#### Follow-Ups

In the cued recall task performed by participants during follow-ups on average 23.18 out of 30 possible correct responses were given over all three sessions. In 12% of all sessions performances reached a high accuracy level between 28 and 30 correct responses (Figure 4). The reduced LMEM for the cued recall data included a by-subject random intercept ($X_{\left(3\right)}^{2}$ = 15.99, *p* < 0.01) as random effects structure. For the fixed effects structure none of the fixed effects of interest (age, stimulation method, and their interaction) significantly increased the model fit. We included the covariates gender ($X_{\left(1\right)}^{2}$ = 9.41, *p* < 0.01), session performance ($X_{\left(1\right)}^{2}$ = 20.19, *p* < 0.001), and follow-up ($X_{\left(2\right)}^{2}$ = 25.97, *p* < 0.001) in the final model. The fixed effect structure explained 35.42% of the variance (marginal *R*^2^) and total variance explained by fixed and random effects structure was 69.79% (conditional *R*^2^). The LMEM revealed a significant main effect of gender (*F*_(1,25)_ = 15.21, *p* < 0.001), follow-up sequence (*F*_(2,55)_ = 13.97, *p* < 0.001), and preceding session performance (*F*_(1,78)_ = 22.96, *p* < 0.001). *Post hoc* evaluation with conditional *t*-tests revealed a significant performance decrease over the follow-up sequences (follow-up two: *β* = −1.32, 95%-CI: (−2.27, −0.37), *t*_(53)_ = −2.7, *p* < 0.001; follow-up three: *β* = −2.68, 95%-CI: (−3.68, −1.7), *t*_(56)_ = −5.3, *p* < 0.001] Women performed significantly better than men [*β* = 3.3, 95%-CI: (1.65, 4.94), *t*_(25)_ = 3.9, *p* < 0.001].

### Recognition

#### Sessions

**Figure 6:**
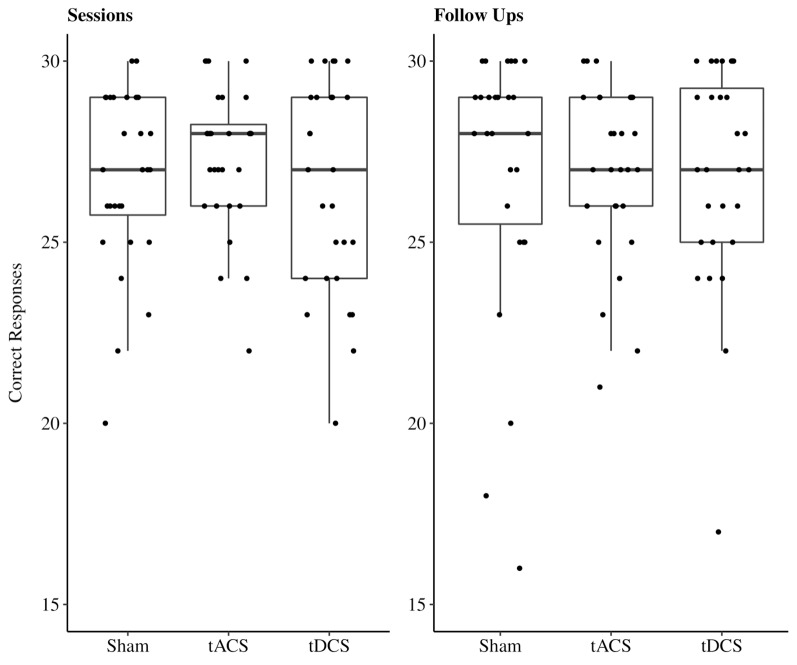
Boxplots with individual data points of sum of correct responses for the recognition task jittered and divided by stimulation method. Abbreviations: tACS, alternating current stimulation; tDCS, direct current stimulation; Sham, control condition.

In the recognition task, on average 26.88 out of 30 possible correct responses were given over all three sessions. In 48% of all sessions performances reached a high accuracy level between 28 and 30 correct responses (Figure 6). Due to these ceiling effects, data should be interpreted with caution. The reduced LMEM for the recognition data included a by-subject random intercept ($X_{\left(1\right)}^{2}$ = 11.52, *p* < 0.001) as random effects structure. For the fixed effects structure only the fixed effects of interest age ($X_{\left(3\right)}^{2}$ = 9.23, *p* < 0.05) significantly increased the model fit, stimulation had no influence on recognition performance during the sessions. Additionally, we included the covariate paired associates baseline performance ($X_{\left(1\right)}^{2}$ = 7.94, *p* < 0.01). The fixed effect structure of the final model explained 33% of the variance (marginal *R*^2^) and total variance explained by fixed and random effects structure of the model was 58.42% (conditional *R*^2^). The LMEM revealed a significantly decreasing task performance with increasing age [*β* = −0.75, 95%-CI: (−1.34, −0.16), *t*_(25)_ = −2.4, *p* < 0.05]. There was a significant effect for paired associates baseline performance on recognition performance [*β* = 1.07, 95%-CI: (0.48, 1.66), *t*_(25)_ = 3.5, *p* < 0.01].

#### Follow-Ups

In the recognition task at follow-ups, on average 27.21 out of 30 possible correct responses were given over all three sessions. In 51% of all sessions performances reached a high accuracy level between 28 and 30 correct responses (Figure 6). As stated above, data should be interpreted with caution due to the ceiling effects. The reduced LMEM for the follow up recognition data included a by-subject random slope ($X_{\left(2\right)}^{2}$ = 18.95, *p* < 0.001) and intercept ($X_{\left(3\right)}^{2}$ = 20.14, *p* < 0.001) as random effects structure. None of the fixed effects of interest increased the model fit, neither stimulation nor age had a significant influence on recognition performance during the sessions. We included the covariates paired associates baseline performance ($X_{\left(1\right)}^{2}$ = 3.67, *p* = 0.05), years of education ($X_{\left(1\right)}^{2}$ = 5.37, *p* < 0.05), and preceding session performance ($X_{\left(2\right)}^{2}$ = 22.46, *p* < 0.01) in the fixed effects structure of our final model. The fixed effect structure explained 61% of the variance (marginal *R*^2^) and total variance explained by fixed and random effects structure of the model was 64% (conditional *R*^2^). The LMEM revealed that preceding session performance had a significant effects on follow-up recognition performance (*β* = 1.67, 95%-CI: [1.06, 2.26], *t*_(29)_ = 5.6, *p* < 0.001). Additionally, number of educational years had a significant influence on recognition performance during follow-ups (*β* = 0.57, 95%-CI: [0.11, 1.01], *t*_(21)_ = 2.53, *p* < 0.05).

### Blinding and Adverse Effects Ratings

The blinding procedure was successful, participants rated on chance level ($X_{\left(2\right)}^{2}$ = 3.4, *p* = 0.18). Due to a technical problem with the online survey at the beginning of data collection, four out of a total of 84 possible ratings are missing from our data set. Generally, stimulation was tolerated well. The most commonly reported side effects were erythema (32%), tingling (26%) and itching (24%). Only the reporting frequency of itching significantly differed between stimulation conditions ($X_{\left(2\right)}^{2}$ = 8, *p* < 0.05, Table 2). Directly comparing stimulation conditions yielded a significant difference for tDCS compared to tACS but not for the real conditions compared to sham (tACS-tDCS: $X_{\left(1\right)}^{2}$ = 6.41, *p* = 0.01; tACS-sham: $X_{\left(1\right)}^{2}$ = 2.12, *p* = 0.15; tDCS-sham: $X_{\left(1\right)}^{2}$ = 0.74, *p* = 0.39).

**Table 2:** Frequency of adverse effect ratings and correct/incorrect guessing for each stimulation condition.

	tACS	tDCS	Sham
Headache	1	2	1
Neck ache	1	0	1
Scalp ache	0	2	1
Tingling	6	11	5
Itching	2	11	7
Erythema	7	12	8
Burning	5	8	4
Fatigue	7	5	4
Loss of concentration	6	3	4
Correct guess	18	21	14
Incorrect guess	9	6	12

*Note: rating sums are shown. tACS, transcranial alternating current stimulation; tDCS, transcranial direct current stimulation; Sham, control condition*.

## Discussion

The present study investigated whether anodal tDCS and theta tACS applied over the left VLPFC would improve associative memory performance in healthy elderly adults. We expected that both stimulation methods would increase associative memory performance by influencing the large-scale functional network connecting prefrontal and medial temporal lobe regions that underlies associative memory encoding. Overall, neither tDCS nor tACS significantly improved memory performance in our subjects. However, the interaction of age and stimulation method showed a significant effect on the outcome of the cued recall task during the sessions. Further exploratory analysis revealed that with increasing age participants performed significantly worse under sham, while there were no significant differences under neither tACS nor tDCS. Further, the comparison of fixed age effects between stimulation methods revealed a significant difference between tACS and sham, but no significant difference between tDCS and sham.

### No Effect of tDCS on Associative Memory Performance

We aimed to influence this network by applying anodal tDCS over the left PFC. With our study design, collecting repeated measurements of a sufficiently large sample, we should have been able to detect the small enhancing effect that tDCS reportedly has on cognitive performance in healthy older adults (*d* = 0.42; Hsu et al., 2015). To our surprise, we did not find any effects. There are several possible explanations for this. We placed anode and cathode on contralateral frontal cortex hemispheres, therefore the current applied is broadly distributed bilaterally. However, this lateral setup of electrodes narrows the current distribution to the frontal cortex (Neuling et al., 2012). Furthermore, we aimed to enhance stimulation focality by using a bigger cathode on the right supraorbital area and a smaller anode over the left VLPFC. This stimulation protocol has repeatedly proven to be successful in enhancing the cognitive performance of healthy older adults (Meinzer et al., 2013; Antonenko et al., 2018, 2019). Importantly, the PFC is a core region of compensatory mechanisms in aging (Sala-Llonch et al., 2015). Concerning the on- and offline effects of tDCS, the optimal time window might differ between young and older healthy adults. A direct comparison of anodal tDCS effects on corticospinal excitability showed that the excitatory effects do not differ in their quality but in their timing: older adults showed a delayed response to tDCS only after 30 min, while young adults’ motor evoked response was immediately influenced by the stimulation (Fujiyama et al., 2014). Thus, tDCS effects on associative memory performance in healthy older adults might be highly variable due to the complexity of underlying neurophysiological mechanisms and the subtle influence of the weak current intensities used. Additionally, tDCS effectiveness varies due to inter-individual differences in susceptibility to the stimulation (Opitz et al., 2015). In our current study we might have missed the optimal time window of tDCS susceptibility during the associative memory encoding processes in our studied group of healthy older participants.

### Interaction of Age and tACS on Associative Memory Performance

We aimed to influence ongoing brain rhythms in the theta frequency range by applying sinusoidal tACS at 5 Hz over the VLPFC during memory encoding. Synchronization of theta networks underlies successful episodic memory encoding with prominent network hubs being located in frontal, temporal and medial temporal cortices (Klimesch, 1999; Solomon et al., 2017). Information binding processes needed for associative memory formation have been causally linked to theta synchronization (Clouter et al., 2017). Poorer-performing older adults showed a significant reduction of theta power in the left inferior frontal gyrus in relation to young adults comparing associative memory performance (Crespo-Garcia et al., 2012). The authors suggested that mainly neural inefficiency of regions in the left VLPFC results in poorer encoding performance of older adults. In our study, only the oldest of the participants benefited from tACS compared to sham. This effect might be explained by a widespread network synchronization originating from neurons in the VLPFC based on entrainment to the oscillatory current of theta tACS. Only with increasing age, frontal neurons become increasingly inefficient and thus susceptible to entrainment by an external current.

Studies investigating theta tACS effects in young adults showed the technique’s feasibility to improve cognitive performance (Polanía et al., 2012; Jaušovec and Jaušovec, 2014). Studies applying tACS in healthy older adults are scarce. However, one recent publication elegantly showed that on one hand the global communication underlying working memory processes are directed by the PFC *via* long-range frontotemporal theta synchronization in young adults, while older adults’ PFC region seems to be insufficiently active causing a decrease in working memory performance. On the other hand, high definition tACS at the individual theta band frequency applied in-phase over the left prefrontal and temporal cortices improved working memory performance in the older adults and reinstated the information flow between prefrontal and temporal regions by reintroducing the frontotemporal theta phase synchronization seen in younger adults (Reinhart and Nguyen, 2019).

To conclude, long-range communication processes become progressively insufficient with increasing age. Possibly, the oscillatory current applied by tACS reinforces successful communication processes between widespread brain regions. We applied sinusoidal theta at 5 Hz in all subjects. Most likely the preset frequency limited stimulation effects to the oldest of our subjects with the least efficient prefrontal theta phase synchronization. Future studies should take care to further optimize stimulation parameters, e.g., by using individual frequency ranges for stimulation protocols.

### Tolerability of Transcranial Electric Current Stimulation in Healthy Older Adults

The threshold for pain perception increases with age, except for the sensations induced by electrical stimulation (Lautenbacher et al., 2017). Still, the stimulation protocol was well tolerated by our elderly participants. The most commonly reported side effects were erythema, tingling, and itching. Only in the tDCS condition itching was reported significantly more often compared to the other conditions. Nevertheless, the blinding procedure was successful. Since all of our participants were novices to NIBS they most likely could not differentiate the time-limited sensations of the ramp-up phase during sham from the longer-lasting sensations of the real stimulation protocols. Compared to tDCS, tACS applied at 1 mA elicited less reporting of any side effects in our participants. The most frequently reported side effect of tACS was fatigue, but not reported significantly more often than in the other conditions. We conclude that 2 mA tDCS and 1 mA tACS applied over 20 min are both safe and well-tolerated stimulation protocols in healthy older adults (Bikson et al., 2016; Matsumoto and Ugawa, 2017).

### Limitations

One limitation of the current study is the limited task difficulty of the associative memory task. Especially for the recognition task, clear ceiling effects were reached by our participants. This has limited our ability to detect any stimulation effects on recognition performance since a medium-to-difficult level is better suited to pick up on the subtle influence of stimulation on memory performance (Hsu et al., 2016). Additionally, even a subset of only 18% of a sample reaching ceiling effects during one occasion of a study can already result in biased parameter estimation of statistical models (Wang et al., 2009). However, we were most interested in the cued recall performance, since we hypothesized that only this mode of memory retrieval relies on an interaction between widespread neocortical and medial temporal lobe regions underlying episodic memory performance (Henke, 2010). In contrast, the recognition task is more restricted to activating neocortical regions and mainly served as a control for successful encoding in our study.

Due to the crossover design, another limitation of our current study is possible carryover effects between stimulation conditions. Although we had a minimum of 72 h in between experimental sessions, NIBS techniques have been hypothesized to directly influence longer-lasting mechanisms of synaptic plasticity, provoking interaction effects between stimulation conditions in our study design (Cirillo et al., 2017). We have randomized order of stimulation methods over all the participants. Additionally, follow-up assessments 24 h after experimental sessions revealed no stable overnight effects for tDCS or tACS in our participants.

Although we conducted an *a priori* power calculation based on the average effect size estimated by a meta-analysis of NIBS studies in healthy and pathological aging (Hsu et al., 2015) the power of our study might still not have been extensive enough. Meta-analyses provide better estimates of true effect size, but these estimates might still be inflated due to different biases (e.g., publication bias; Button et al., 2013). Thus, the result of our estimated sample size might have been overly optimistic.

A further limitation of our study is the absence of neurophysiological measures. Simultaneous collection of neurophysiological correlates using techniques like fMRI or EEG might reveal interaction effects of NIBS and possibly compensatory changes of functional activation and connectivity patterns not becoming evident in purely cognitive outcomes (Meinzer et al., 2013; Marceglia et al., 2016). An example of a possible interaction effect is given by the cognitive reserve hypothesis: older adults with higher cognitive reserve resist beginning age-related pathologies longer by functional compensation mechanisms and thus might be less susceptible to NIBS techniques (Cespón et al., 2018). Future studies investigating developmental dynamics and their interaction with NIBS should use EEG, fMRI or similar techniques to uncover stimulation-induced neurophysiological changes.

## Conclusion

Potential memory-enhancing effects of tES methods rely on optimal stimulation setups. Our mixed results show that reliable and reproducible stimulation effects on memory performance in healthy older adults are not yet easily achieved. Ideally, future studies trying to efficiently enhance associative memory performance in aging should identify measurable neurophysiological correlates that define optimal time windows of individual responsiveness to tES. Our findings indicate the potential of theta tACS to positively influence the widespread network communication needed to maintain successful associative memory formation with increasing age.

## Data Availability Statement

The datasets generated for this study are available on request to the corresponding author.

## Ethics Statement

The studies involving human participants were reviewed and approved by Kantonale Ethikkommission Bern (KEK). The patients/participants provided their written informed consent to participate in this study.

## Author Contributions

KK and SK designed research. KK performed research and drafted the article. KK and PW analyzed data. SK, JP, and PW revised the article critically.

## Conflict of Interest

The authors declare that the research was conducted in the absence of any commercial or financial relationships that could be construed as a potential conflict of interest.

## Footnotes

^1^https://www.berufsberatung.ch
