# Supplementary material for: Transcranial Electric Current Stimulation During Associative Memory Encoding: Comparing tACS and tDCS Effects in Healthy Aging
Source: Front Aging Neurosci. 2020 Mar 17;12:66. doi: 10.3389/fnagi.2020.00066 (PMC7090128; doi:10.3389/fnagi.2020.00066)
Supplement: Supplementary file 1 [file Image_1.PDF]

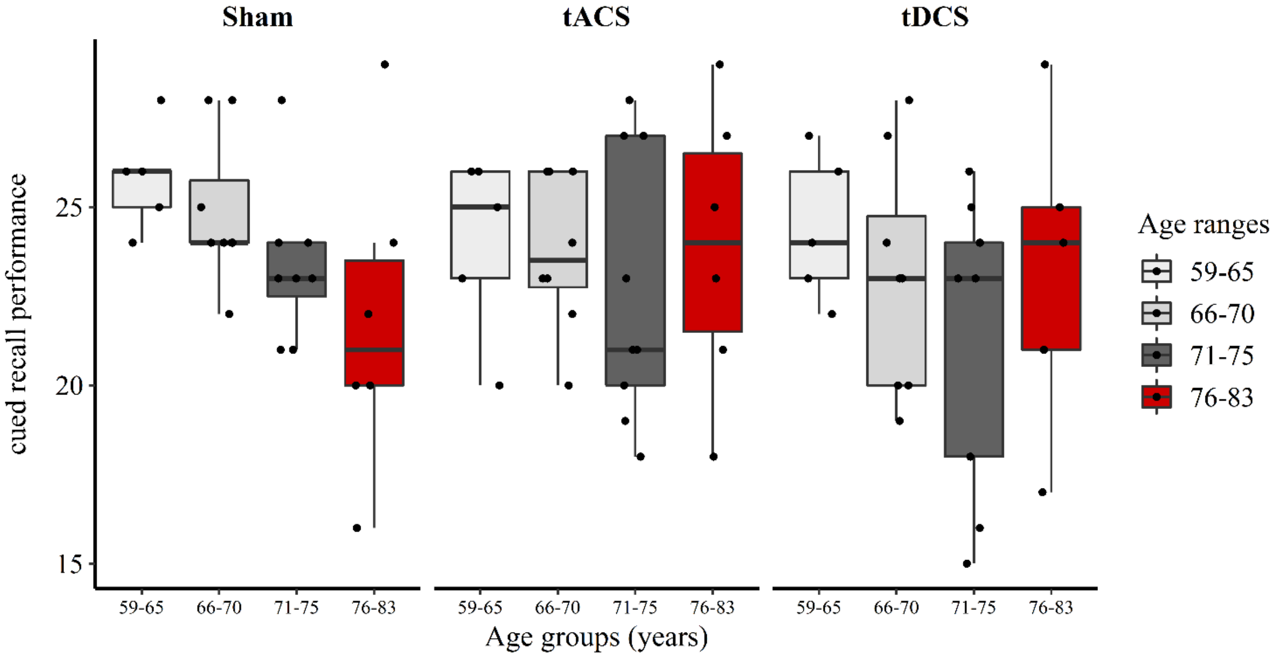

Supplementary Figure 1. Boxplots with individual data points of sum of correct responses for the cued recall task jittered and divided by age groups shown for each stimulation method. Abbreviations: tACS, alternating current stimulation; tDCS, direct current stimulation; Sham, control condition.
