# Supplementary material for: Transcranial Electric Current Stimulation During Associative Memory Encoding: Comparing tACS and tDCS Effects in Healthy Aging
Source: Front Aging Neurosci. 2020 Mar 17;12:66. doi: 10.3389/fnagi.2020.00066 (PMC7090128; doi:10.3389/fnagi.2020.00066)
Supplement: Supplementary file 2 [file Image_2.PDF]

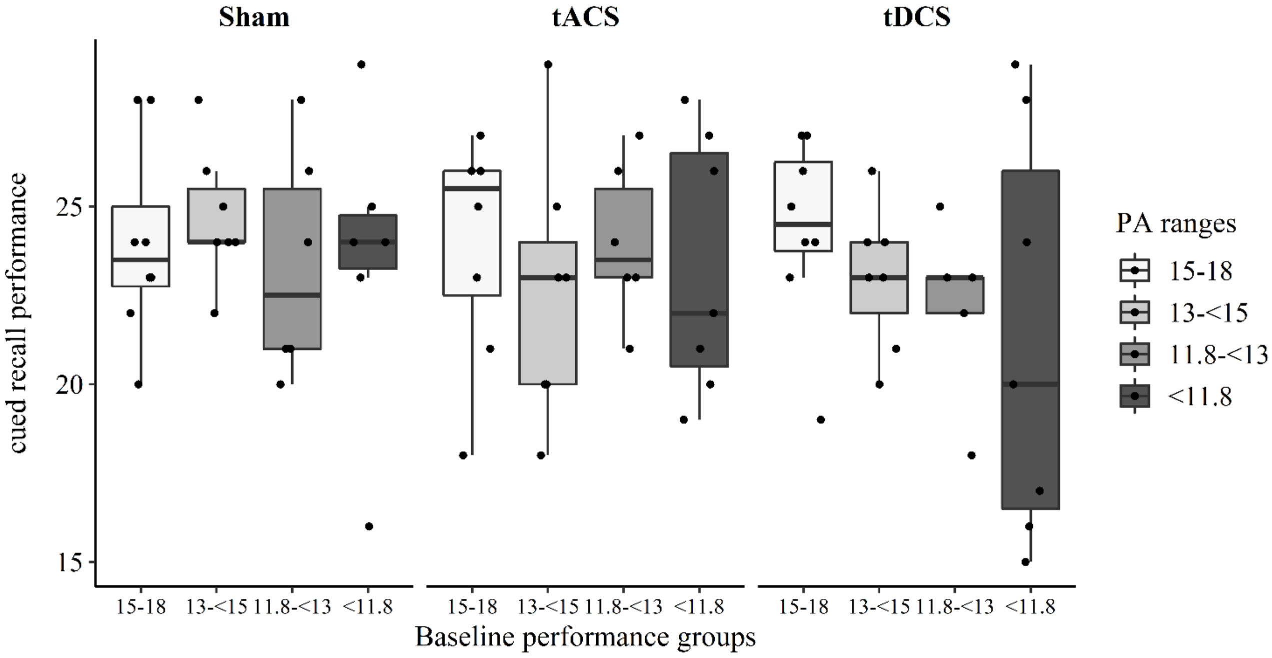

Supplementary Figure 2. Boxplots with individual data points of sum of correct responses for the cued recall task jittered and divided by baseline performance groups shown for each stimulation method. Abbreviations: tACS, alternating current stimulation; tDCS, direct current stimulation; Sham, control condition; PA, paired associates test delayed recall (Petermann and Lepach, 2012; Spaan, 2016).
