# Supplementary material for: Transcranial Electric Current Stimulation During Associative Memory Encoding: Comparing tACS and tDCS Effects in Healthy Aging
Source: Front Aging Neurosci. 2020 Mar 17;12:66. doi: 10.3389/fnagi.2020.00066 (PMC7090128; doi:10.3389/fnagi.2020.00066)
Supplement: Supplementary file 3 [file Table_1.DOCX]

Supplementary Material

# Supplementary Figures and Tables

## Supplementary Tables

**Table 1.** Overview of the stimuli used for the face-occupation associative memory task and their English translation.

| Ausbildung/ Education | | | |
| --- | --- | --- | --- |
| **Universität** | **University degree** | **Lehre** | **Apprenticeship** |
| Maschineningenieur | mechanical engineer | Coiffeur | hair dresser |
| Psychotherapeut | psychotherapist | Zimmermann | carpenter |
| Archäologe | archeologist | Uhrmacher | watchmaker |
| Sportwissenschaftler | sport scientist | Seilbahner | ropeway technican |
| Kunsthistoriker | art historian | Heizungsinstallateur | heating engineer |
| Hausarzt | family doctor | Kosmetiker | beautician |
| Biologe | biologist | Pferdewart | groom (horses) |
| Politikwissenschaftler | political scientist | Glaser | glazier |
| Neuropsychologe | neuropsychologist | Fahrzeugschlosser | auto mechanic |
| Pharmazeut | pharmacist | Zugführer | conductor |
| Theologe | theologist | Winzer | wine grower |
| Physiker | physicist | Carosserielackierer | auto varnisher |
| Tierarzt | veterinarian | Floristin | florist |
| Biochemiker | biochemist | Lastwagenfahrer | truck driver |
| Kommunikations-  wissenschaftler | communications scientist | Flugbegleiter | steward/ess |
| **Maximales Einkommen/ Maximum Income** | | | |
| **Überdurchschnittlich** | **Above average** | **Durchschnittlich** | **Average** |
| Rechtsanwalt | lawyer | Fleischfachmann | butcher |
| Zahnarzt | dentist | Strassenbauer | road builder |
| Bezirksanwalt | district attorney | Büroassistent | office assistant |
| Finanzanalyst | financial analyst | Fahrradmechaniker | bike mechanic |
| Radiologe | radiologist | Busfahrer | bus driver |
| Architekt | architect | Maurer | bricklayer |
| Patentanwalt | patent attorney | Bühnentänzerin | stage dancer |
| Professor | professor | Kaminfeger | chimney sweep |
| Notar | notary | Verkäufer | sales assistant |
| Bankjurist | financial lawyer | Hausmeister | janitor |
| Diplomat | diplomat | Maler | painter |
| Chirurg | surgeon | Schneider | tailor |
| Richter | judge | Buchhändler | bookseller |
| Betriebswirtschafter | business manager | Dentalassistent | dental hygienist |
| Staatsanwalt | public prosecutor | Tierpfleger | zoo-keeper |
| **Schwerpunkt der Tätigkeit/ Focus of Labor Activity** | | | |
| **Geistig** | **Mental** | **Manuell** | **Manual** |
| Mathematiker | mathematican | Gärtner | gardener |
| Meterologe | meterologist | Koch | chef |
| Linguist | linguist | Spengler | plumber |
| Kriminologe | criminologist | Parkettleger | parquet recliner |
| Wissenschaftlicher Bibliothekar | scientific librarian | Geigenbauer | violin maker |
| Dolmetscher | interpreter | Bäcker | baker |
| Steuerberater | tax consultant | Steinmetz | stonemason |
| Journalist | journalist | Schreiner | joiner |
| Philosoph | philosopher | Schuhmacher | shoemaker |
| Versicherungsmathe-matiker | insurance mathematican | Forstwart | forester |
| Ökonom | economist | Gebäudereiniger | cleaner |
| Historiker | historian | Gleisbauer | track layer |
| Musikwissenschaftler | musicologist | Sanitärinstallateur | plumber |
| Wirtschaftsprüfer | accountant | Dachdecker | roofer |
| Statistiker | statistician | Landwirt | farmer |
