# Supplementary material for: Transcranial Electric Current Stimulation During Associative Memory Encoding: Comparing tACS and tDCS Effects in Healthy Aging
Source: Front Aging Neurosci. 2020 Mar 17;12:66. doi: 10.3389/fnagi.2020.00066 (PMC7090128; doi:10.3389/fnagi.2020.00066)
Supplement: Supplementary file 4 [file Table_2.DOCX]

Supplementary Material

# Supplementary Figures and Tables

## Supplementary Tables

**Table 2.** T-test results using Satterthwaite’s approximation for the reduced linear mixed models fit by restricted maximum likelihood (REML) for cued recall performances during sessions and follow-ups (results are shown for reference levels *sham* and *session/follow-up 1).*

| **Sessions** |  |  |  |
| --- | --- | --- | --- |
| Predictor | beta (*SE*) | 95%-CI | *t* (df) |
| (Intercept) | 23.5 (0.63) | [22.31, 24.7] | 37.04 (63) |
| Education | 0.96 (0.47) | [0.06, 1.87] | 2.04 (25) |
| Session 2 | - 0.72 (0.55) | [- 1.75, 0.33] | - 1.3 (48) |
| Session 3 | 1.31 (0.55) | [0.27, 2.35] | 2.36 (48) |
| Age | -1.58 (0.56) | [- 2.64, - 0.52] | - 2.8 (47) |
| ACS | -0.28 (0.54) | [- 1.3, 0.74] | -0.51 (48) |
| DCS | -0.93 (0.55) | [- 1.97, 0.097] | - 1.7 (48) |
| Age:ACS | 1.18 (0.54) | [0.16, 2.2] | 2.17 (48) |
| Age:DCS | 1.05 (0.57) | [- 0.02, 2.12] | 1.85 (48) |
| *N*= 82, *REML*= 374, random intercept for participant with *SD*= 2.16 and residual with *SD*= 1.99 | | | |
| **Follow ups** |  |  |  |
| Predictor | beta (*SE*) | 95%-CI | *t* (df) |
| (Intercept) | 22.63 (0.7) | [21.28, 23.98] | 32.4 (36) |
| Follow-Up 2 | -1.32 (0.49) | [- 2.27, - 0.37] | -2.7 (53) |
| Follow- Up 3 | -2.68 (0.51) | [- 3.68, - 1.7] | -5.3 (56) |
| Session performance | 1.47 (0.31) | [0.88, 2.08] | 4.8 (79) |
| Gender (female) | 3.3 (0.85) | [1.65, 4.94] | 3.9 (25) |
| *N*= 84, *REML*=374.5, random intercept for participant with *SD*= 1.95 and residual with *SD*= 1.83 | | | |

*Note: ACS, transcranial alternating current stimulation; CI, confidence interval; DCS, transcranial*

*direct current stimulation; Df, degrees of freedom; N, number of observations; SD, standard deviation;*

*SE, standard error.*
