# Supplementary material for: Transcranial Electric Current Stimulation During Associative Memory Encoding: Comparing tACS and tDCS Effects in Healthy Aging
Source: Front Aging Neurosci. 2020 Mar 17;12:66. doi: 10.3389/fnagi.2020.00066 (PMC7090128; doi:10.3389/fnagi.2020.00066)
Supplement: Supplementary file 5 [file Table_3.DOCX]

Supplementary Material

# Supplementary Figures and Tables

## Supplementary Tables

**Table 3.** T-test results using Satterthwaite’s approximation for the reduced linear mixed models fit by restricted maximum likelihood (REML) for recognition performances during sessions and follow-ups (results are shown for reference level *session/follow-up 1).*

| **Sessions** |  |  |  |
| --- | --- | --- | --- |
| Predictor | beta (*SE*) | 95%-CI | *t* (df) |
| (Intercept) | 26.88 (0.3) | [26.31, 27.45] | 90.5 (25) |
| PA delayed recall | 1.07 (0.31) | [0.48, 1.66] | 3.5 (25) |
| Age | - 0.75 (0.31) | [- 1.34, - 0.16] | - 2.43 (25) |
| *N*= 84, *REML*=347.1, random intercept for subject with *SD* = 1.27 and residual with *SD* = 1.62 | | | |
| **Follow ups** |  |  |  |
| Predictor | beta (*SE*) | 95%-CI | *t* (df) |
| (Intercept) | 27.21 (0.22) | [26.77, 27.67] | 122.32 (16.77) |
| PA delayed recall | 0.43 (0.22) | [- 0.004, 0.87] | 1.93 (18.46) |
| Education | 0.57 (0.22) | [0.11, 1.01] | 2.53 (20.76) |
| Session performance | 1.67 (0.3) | [1.06, 2.26] | 5.6 (29.44) |
| *N*= 83, *REML*=327.4, random intercepts for participant with *SD* = 0.46 and residual with *SD* = 1.54, random slope with *SD* = 0.97 | | | |

*Note: CI, confidence interval; df, degrees of freedom; N, number of observations; PA, paired associates; SD, standard deviation; SE, standard error.*
